# PCNN Model Guided by Saliency Mechanism for Image Fusion in Transform Domain

**DOI:** 10.3390/s23052488

**Published:** 2023-02-23

**Authors:** Liqun Liu, Jiuyuan Huo

**Affiliations:** 1 College of Information Science and Technology, Gansu Agricultural University, Lanzhou 730070, China; 2 School of Electronic and Information Engineering, Lanzhou Jiaotong University, Lanzhou 730070, China

**Keywords:** significance function, first-order Markov, mutual information, pulse coupled neural network, image fusion

## Abstract

In heterogeneous image fusion problems, different imaging mechanisms have always existed between time-of-flight and visible light heterogeneous images which are collected by binocular acquisition systems in orchard environments. Determining how to enhance the fusion quality is key to the solution. A shortcoming of the pulse coupled neural network model is that parameters are limited by manual experience settings and cannot be terminated adaptively. The limitations are obvious during the ignition process, and include ignoring the impact of image changes and fluctuations on the results, pixel artifacts, area blurring, and the occurrence of unclear edges. Aiming at these problems, an image fusion method in a pulse coupled neural network transform domain guided by a saliency mechanism is proposed. A non-subsampled shearlet transform is used to decompose the accurately registered image; the time-of-flight low-frequency component, after multiple lighting segmentation using a pulse coupled neural network, is simplified to a first-order Markov situation. The significance function is defined as first-order Markov mutual information to measure the termination condition. A new momentum-driven multi-objective artificial bee colony algorithm is used to optimize the parameters of the link channel feedback term, link strength, and dynamic threshold attenuation factor. The low-frequency components of time-of-flight and color images, after multiple lighting segmentation using a pulse coupled neural network, are fused using the weighted average rule. The high-frequency components are fused using improved bilateral filters. The results show that the proposed algorithm has the best fusion effect on the time-of-flight confidence image and the corresponding visible light image collected in the natural scene, according to nine objective image evaluation indicators. It is suitable for the heterogeneous image fusion of complex orchard environments in natural landscapes.

## 1. Introduction

Automatic apple fruit picking in natural environments can reduce the intensity of heavy manual labor, which is an inevitable choice for modern agriculture [1]. The natural light in northwest China is strong, and the visible light images collected in the natural environment are vulnerable to the influences of changing light and complex backgrounds, so the recognition effect lacks some robustness [2]. In the complex environment of orchard operations, the most potential in vision research on picking robots lies in the technology of heterogeneous image fusion (IF) between time-of-flight (ToF) images and visible light images. The collected images have a variety of different attributes, including light invariance, spatial hierarchy, infrared perception, reliability of discrimination data, etc. [2]. The image is indirectly generated from the depth information, which can reflect the near–far relationship and infrared reflection characteristics of different objects in the scene, and the effect is not affected by light changes [2]. Image fusion generates a new information processing process that interprets the scene from a different source image which cannot be obtained from the information obtained by a single sensor [2,3]. Determining how to fuse ToF images and visible light images with different wavelength ranges and imaging mechanisms with high quality is currently a topic of great interest in image fusion research.

A non-subsampled shearlet transform (NSST) is a multi-scale, multi-directional, translation-invariant transform domain image decomposition method, which is widely used in image fusion [4]. An NSST shearlet wave transform avoids the down-sampling operation, and has the characteristics of translation invariance, simple operation, low time complexity, etc. [5]. Compared with wavelet transforms such as the discrete wavelet transform (DWT), stationary wavelet transform (SWT), discrete cosine transform (DCT), curvelet transform, and contourlet transform, an NSST has a good effect on searching for edges and contours. There are large numbers of deep neural network layers in deep learning methods. This characteristic could lead to low efficiency and a high cost. The advantage of an NSST is that it can fully fuse the source image information, and the fused image has good correlation coefficient and information entropy, which is more suitable for the situation where the image background in the natural orchard environment is complex, and the contour and image texture information need to be fused at the same time.

Related works are summarized as follows: A pulse coupled neural network (PCNN) is a neural network model established by simulating the activities of visual nerve cells in the cerebral cortex. Similar pattern features are classified into categories based on the principles of similarity clustering and capture characteristics [6]. In terms of image fusion in a transform domain, Cheng et al. used an adaptive dual-channel pulse coupled neural network with triple connection strength in the local non-down-sampled shear wave transform domain to solve the spectral difference between infrared and visible light [7]. Panigrahy et al. proposed a new medical fusion method in a non-down-sampled shear wave transform domain based on a weighted parameter adaptive dual channel PCNN [8]. In terms of image fusion in saliency attention models, Liu et al. proposed a saliency detection model that combines a global saliency map with a local saliency map [9]. Yang et al. designed a new fuzzy logic rule based on global saliency measurements to fuse the details extracted from panchromatic images with high spatial resolution and multispectral images with low spatial resolution [10]. Li et al. used the segmentation-driven low-rank matrix recovery model to detect the significance of each individual image in the image set to highlight the regions with sparse features in each image [11]. In terms of the optimization of image fusion parameters, Zhu et al. applied PCNN parameters to infrared and visible image fusion through quantum-behavior particle swarm optimization improvement [12]. Huang et al. used an NSCT to independently decompose the intensity hue saturation of the image, a PCNN to fuse high-frequency sub-band images and low-frequency images, and a hybrid leapfrog algorithm to optimize PCNN parameters [13]. Dharini et al. proposed a nature-inspired optimal feature selection method using ant colony optimization to reduce the complexity of the PCNN fusion of infrared and visible images [14]. In the research of overexposure problems with concern to the ongoing climate change-related environmental changes over mountainous areas, Muhuri et al. used polarization fraction variation with temporal RADARSAT-2 C-Band full-polarimetric to study SAR Data [15]. Raskar et al. introduced a novel technique to allow a user to interact with projected information and to update the projected information [16].

A PCNN classifies similar pattern features into categories based on the principles of similarity aggregation and capture characteristics. The segmentation combination has the advantages of the grayscale aggregation lighting mechanism and the same grayscale attribute priority lighting. This is consistent with the basic idea of cluster analysis. Qiu et al. proposed a new density peaks-based clustering method, called clustering with local density peaks-based minimum spanning tree [17]. Huang et al. proposed new adaptive spatial regularization for the representation coefficients to improve the robustness of the model to noise [18]. Huang et al. proposed ultra-scalable spectral clustering and ultra-scalable ensemble clustering methods [19].

Although scholars have studied the optimization and improvement of PCNN parameters, there are still cases of pixel artifacts, region blurring and unclear edges due to ignoring the impact of image changes and fluctuations on the results during the ignition process.

This paper introduces the concept of entropy [20] in information theory and proposes a PCNN model guided by a saliency mechanism (SMPCNN). The ToF low-frequency component after multiple lighting segmentation using PCNN is simplified into a first-order Markov situation, and the significance function is defined as first-order Markov mutual information. On this basis, a PCNN model guided by a saliency mechanism for image fusion in transform domain (NSST-SMPCNN) is proposed to fuse ToF and visible light heterogeneous images collected by a binocular acquisition system in an orchard environment.

We summarize our main contributions below.

First, we aim to solve the following existing problems:

1. The traditional method of space domain fusion is to create a fusion model in the image gray space, which has the disadvantage that it is not easy to find the source image texture and boundary features.
2. A PCNN model has the defects of parameter experience setting, unadaptive termination, and easy over-segmentation. In the ignition process, it ignores the impact of image change fluctuation on the results, resulting in pixel artifacts, area blurring, and unclear edges.
3. The differences in imaging mechanisms between ToF and visible light heterogeneous images collected by a binocular acquisition system in an orchard environment lead to the problem of low fusion quality.

Second, the innovations and novelties of this paper are as follows:

1. A PCNN model guided by a saliency mechanism is proposed and applied to the fusion of ToF and visible light heterogeneous images collected by a binocular acquisition system in an orchard environment.
2. The ToF low-frequency component is simplified after multiple lighting segmentation using a PCNN into a first-order Markov situation. The significance function is defined as first-order Markov mutual information.
3. The significance function is used as the termination condition of a PCNN model iteration, and Kullback–Leibler (KL) divergence is used to measure the dynamic threshold amplification coefficient of the PCNN model.
4. A new momentum-driven multi-objective artificial bee colony algorithm is proposed to optimize the parameters of link channel feedback, link strength, and dynamic threshold attenuation factor. The momentum update strategy of employing bees and observing bees is used. The grid density construction is used to ensure that the optimal solution distribution is not too dense. The absolute value of the difference between the grid index values of the same dimension of the nondominated solution is used as the deletion selection probability of the nondominated solution to construct the optimal solution set. Cross entropy (CE) and mutual information (MI), two image fusion quality evaluation functions, are selected as multi-objective fitness functions.
5. The low-frequency components of ToF and color image after multiple lighting segmentation using a PCNN are fused using the weighted average rule, and the high-frequency components are fused using improved bilateral filters.

Three, the advantage of our work is as follows: the proposed NSST-SMPCNN method combines the saliency mechanism, saliency function, and the PCNN clustering segmentation mechanism, and has the advantages of a grayscale clustering lighting mechanism and the same grayscale attribute first lighting, which is suitable for heterogeneous image fusion in complex orchard environments in Gansu.

The paper structure is summarized as follows: Section 1 contains the introduction and a description of the related works, as well as the highlights and contributions of this paper. Then, basic concept definitions of an NSST and a PCNN are introduced. The proposed definition of significance function is also defined in Section 2. In Section 3, a PCNN model guided by a saliency mechanism is proposed. Then, a PCNN transform domain image fusion method guided by a saliency mechanism is constructed in the Section 4. Lastly, the final section contains a description of the experiment and the conclusions.

## 2. Basic Concept Definition

### 2.1. NSST Transform Domain Decomposition Method

The traditional method of spatial domain fusion is to create a fusion model in the image gray space. The disadvantage of this is that it is difficult to find the texture and boundary features of the source image. The NSST transform domain decomposition method, which is proposed in reference [4], is used to perform the non-subsampled pyramid filter bank (NSP) u-level transformation on the two accurately registered heterogeneous images to obtain one low-frequency sub-band and u high-frequency sub-bands, realizing translation invariance. The high-frequency sub-band is then decomposed into 2*^v^* directional high-frequency sub-bands by shear filter bank (SF) v-level multi-directional decomposition, so as to effectively capture directional information and maintain anisotropy [4]. The decomposed sub-band is the same size as the source image, has high sparsity, and accurately represents the fusion information.

### 2.2. PCNN Lighting Segmentation Mechanism

The PCNN model proposed in reference [6] includes feedback input domain, coupling link domain, and pulse generation domain, which can be described by the mathematical equations shown in Formulas (1)–(5). A PCNN has a feature which classifies similar pattern features into categories based on the principles of similarity aggregation and capture characteristics, and has the mechanism of aggregation and illumination segmentation.

(1) $$F_{ij}(n)=I_{ij}$$

(2) $$L_{ij}(n)=\exp(-\alpha_{L})L_{ij}(n-1)+V_{L}\underset{kl}{\sum }W_{ij,kl}Y_{kl}(n-1)$$

(3) $$U_{ij}(n)=F_{ij}(n)(1+\beta L_{ij}(n))$$

(4) $$\theta_{ij}(n)=\exp(-\alpha_{\theta })\theta_{ij}(n-1)+V_{\theta }Y_{ij}(n)$$

(5) $$Y_{ij}(n)=step(U_{ij}(n)-\theta_{ij}(n))$$

In the formula, *I_ij_* is the external stimulation of neurons, represented by the gray value of the input image; *F_ij_*(*n*) is the feedback input field; *L_ij_*(*n*) is the link input field; *W_ij_*_,*kl*_ refers to the link coefficient; *β* indicates the link strength, which determines the weight of the coupling link channel; *U_ij_*(*n*) is the internal state signal of the model; *θ_ij_* is the dynamic threshold of neurons, *V_θ_* and *V_L_* are the dynamic threshold amplification coefficients, which control the threshold value increased after neuron activation; *α_L_* and *α_θ_* determine the decay rate of the feedback term and the dynamic threshold of the link channel, respectively; *Y_ij_*(*n*) is the pulse output of the current neuron, which is the response result of the comparison between the internal active item and the dynamic threshold in the pulse generator. When *U_ij_*(*n*) > *θ_ij_*(*n*), the ignition condition will be reached and the output *Y_ij_*(*n*) = 1. Step represents a step function, and its output is 0 or 1; n represents the nth neuron in the image.

### 2.3. Proposed Definition of Significance Function

The saliency mechanism originates from the visual attention mechanism (VAM) proposed by Itti and other scholars [21], inspired by the behavior and neuronal structure of early primate visual systems [22]. When the saliency mechanism processes a scene, it automatically processes the regions of interest, and selectively ignores the regions of noninterest. In this paper, a new saliency mechanism to define the significance function is proposed.

**Definition 1.**  *Significance first-order Markov situation.*

The ToF low-frequency component after an NSST decomposition is sent into the PCNN model. The PCNN is divided iteratively many times, showing a dynamic ignition segmentation state. The two ignition segmentation diagrams at the time intervals t and t + 1 are correlated, but independent of the ignition segmentation diagram at the previous time. Therefore, the ignition segmentation diagram at the time interval 2 can be defined as a first-order Markov situation.

**Definition 2.**  *Significance one-step transition probability.*

When the model is in the state $s_{u}$ after ignition segmentation at time t, the probability of model transition to the state $s_{v}$ after ignition segmentation at time t + 1 is defined as the significant one-step transition probability, which is expressed in Formula (6).

(6) $$p_{uv}=p(S_{t+1}=s_{v}\left|S_{t}=s_{u}\right.)=p(s_{v}\left|s_{u}\right.),s_{u},s_{v}\in S$$

**Definition 3.**  *Significant conditional entropy.*

In the significance first-order Markov situation, the average uncertainty of the model when it is transferred to the state $s_{v}\in S$ under any state condition $s_{u}\in S$ is defined as the significance conditional entropy, which is expressed in Formula (7).

(7) $$H(U\left|V\right.)=-\overset{N}{\underset{v=0}{\sum }}p(s_{u},s_{v})\log p(s_{v}\left|s_{u}\right.)$$

**Definition 4.**  *Significance first-order Markov information source entropy.*

The overall uncertainty of the sequence formed by the ignition segmentation map in the significance first-order Markov situation is defined as the significance first-order Markov information source entropy, which is expressed in Formula (8).

(8) $$H(U)=-\overset{N}{\underset{u=0}{\sum }}\overset{N}{\underset{v=0}{\sum }}p(x_{v},s_{u})\log p(x_{v}\left|s_{u}\right.)$$

**Definition 5.**  *Significance first-order Markov mutual information.*

The amount of information transmitted in the model state transition of the ignition segmentation map at different times is defined as the significant first-order Markov mutual information, which is expressed as Formula (9).

(9) $$I(U;V)=H(U)-H(U\left|V\right.)$$

**Definition 6.**  *Significance function.*

The PCNN is divided by multiple iterations. The ignition segmentation graph with two time intervals has significant feature differences, representing the maximum information transmission rate and the maximum amount of mutual information in numerical terms. Because mutual information has a maximum under certain conditions, the significance function is numerically defined as significant first-order Markov mutual information. If Formula (7) and (8) are brought into Formula (9), Formula (10) is formed.

(10) $$I_{saliency}(u,v)=I(U;V)=-\overset{N}{\underset{u=0}{\sum }}\overset{N}{\underset{v=0}{\sum }}p(x_{v},s_{u})\log p(x_{v}\left|s_{u}\right.)+\overset{N}{\underset{v=0}{\sum }}p(s_{u},s_{v})\log p(s_{v}\left|s_{u}\right.)$$

## 3. PCNN Model Guided by Saliency Mechanism

PCNN region segmentation and a saliency mechanism can locate the most interesting object region in the image well. Combining the saliency mechanism, saliency function and PCNN clustering segmentation mechanism, a PCNN model guided by a saliency mechanism is proposed, which has the advantages of a grayscale clustering lighting mechanism and the same grayscale attribute lighting priority, and is suitable for heterogeneous image fusion in complex orchard environments in Gansu.

The PCNN model has certain shortcomings, including that the parameters are limited by manual experience settings and cannot be terminated adaptively, and ignoring the impact of image changes and fluctuations on the results during the ignition process results in pixel artifacts, area blurring, and unclear edges. The iteration termination conditions and dynamic threshold amplification coefficients $V_{\theta }$ and the feedback items of the link channel *α_L_*, link strength *β*, and dynamic threshold attenuation factor *α_θ_* are improved adaptively. A new momentum-driven multi-objective artificial bee colony algorithm (MMOABC) is used for parameter optimization and is applied to the proposed PCNN model guided by a saliency mechanism. The improved SMPCNN model has the characteristics of enhancing the same type of pulse connection, reducing the difficulty of parameter integration, and improving the performance of image segmentation.

### 3.1. Adaptive Iteration Termination Conditions

The traditional PCNN model has the defects of nonadaptive termination and over-segmentation. The authors of [23] used the maximum information entropy as the termination condition, but over-segmentation often occurs when the entropy is at its maximum, and the background with the same gray value will be mistaken for the target area and segmented together.

In this paper, the significance function is used as the criterion for model iteration termination, which is expressed as Equation (11). For a low-frequency ignition segmentation map, the greater the significance of the first-order Markov mutual information, the better the regional consistency.

(11) $$I_{saliency}(u,v)>\delta$$

### 3.2. Adaptive Dynamic Threshold Amplification Coefficient $V_{\theta }$

In the ToF image, the fruit target is often shown as a region with a high gray value and normal distribution. Two ignition segmentation images are used to measure the PCNN dynamic threshold amplification coefficient, which is expressed as Equation (12). The probability distribution $p(s_{u})$ corresponding to the state $s_{u}\in S$, as well as the probability distribution $p(s_{v})$ corresponding to the state $s_{v}\in S$, and the KL divergence of the two states are calculated. This formula is used to measure the similarity between the probability distributions of two ignition segmentation maps. The closer the probability distribution of the two ignition segmentation images is, the smaller the dynamic threshold amplification coefficient is, which will enable the PCNN model to ignite when the target region tends to be stable during continuous iteration.

(12) $$V_{\theta }=D_{KL}(s_{v}\left|\right|s_{u})=\sum_{v=1}^{N}\left[p(s_{v})\log p(s_{v})-p(s_{v})\log p(s_{u})\right]$$

### 3.3. Parameter Optimization of Momentum Driven Multi-Objective Artificial Bee Colony Algorithm

An artificial bee colony algorithm (ABC) [24] is a swarm intelligence optimization algorithm proposed to simulate the characteristics of bee swarms. It has the advantages of strong global optimization ability, few parameters, high accuracy, and strong robustness. However, its optimization strategy has the defects of simplicity and randomness, which make the algorithm premature, cause convergence stagnation, and other problems. In order to accelerate the convergence rate of the artificial bee colony algorithm, the concept of momentum [25,26] in deep learning is introduced, and a new momentum-driven multi-objective artificial bee colony algorithm is proposed to optimize the three parameters. including the feedback from the link channel *α_L_*, link strength *β*, and dynamic threshold attenuation factor *α_θ_*.

#### 3.3.1. Initial Population

The three parameters, including feedback from the link channel *α_L_*, link strength *β*, and dynamic threshold attenuation factor *α_θ_*, are used as the initial population of the momentum-driven multi-objective artificial bee colony algorithm. Random generation of NP food source information $X=\left\{x_{ij}\left|x_{ij}=\left(x_{i1},x_{i2},\cdots ,x_{ij},\cdots ,x_{id}\right),i=1,2,\cdots ,NP;j=1,2,\cdots ,d=3\right.\right\}$ was performed according to Formula (13).

(13) $$x_{ij}=\min_{j}+rand(0,1)\times (\max_{j}-\min_{j})$$

#### 3.3.2. Hiring Bees Momentum Updating Strategy

NP food source information was randomly generated. During a food update evolution, a randomly selected food source $X_{k}=(x_{k1},x_{k2},\cdots ,x_{kd})$ was attached to a hired bee in the bee colony. In the d-dimensional space, the randomly selected jth dimension component $x_{ij}$ of each food source $X_{i}=(x_{i1},x_{i2},\cdots x_{ij},\cdots ,x_{id})$ in the food source information space database was evolved through the following hired bee momentum update strategy, as shown in Equations (14) and (15), to obtain a new food source $X_{i}^{new}=(x_{i1},x_{i2},\cdots ,x_{ij}^{new},\cdots ,x_{id})$. Among them, i,k∈[1,2,⋯,NP],i≠k,j∈[1,2,⋯,d],r∈[−1,1]. In Equations (14) and (15), $a_{ij}$ represents the update step size of the previous update evolution, $a_{ij}^{new}$ represents the update step size obtained after the current momentum update evolution, γ represents momentum, and the value is 0.9.

(14) $${x_{ij}}^{new}=x_{ij}+a_{ij}^{new}$$

(15) $$a_{ij}^{new}=r\times (x_{ij}-x_{kj})+\gamma \times a_{ij}$$

#### 3.3.3. Observation Bees Nesterov Momentum Updating Strategy

In a food update evolution, the selection probability of observation bees was calculated according to Formula (16), and a randomly selected food source $X_{t}=(x_{t1},x_{t2},\cdots ,x_{td})$ was attached to an observation bee in the bee colony. In the d-dimensional space, the randomly selected jth dimension component $x_{ij}$ of each food source $X_{i}=(x_{i1},x_{i2},\cdots x_{ij},\cdots ,x_{id})$ in the food source information space database was evolved through the following observation bees Nesterov momentum updating strategy, as shown in Formulas (17) and (18), to obtain a new food source $X_{i}^{new}=(x_{i1},x_{i2},\cdots ,x_{ij}^{new},\cdots ,x_{id})$. Among them, i,t∈[1,2,⋯,NP],i≠t,j∈[1,2,⋯,d],r∈[−1,1]. In Formulas (17) and (18), $b_{ij}$ represents the update step size of the previous update evolution, $b_{ij}^{new}$ represents the update step size obtained after the current Nesterov momentum update evolution, γ represents momentum, and the value is 0.9. Target=2,j=1,2,⋯,NP.

(16) $$prob_{j}=\sum_{i=1}^{T\arg et}(0.9\times (f_{i}/\max(f_{i}))+0.1)\times (1/T\arg et)$$

(17) $${x_{ij}}^{new}=x_{ij}+b_{ij}^{new}$$

(18) $$b_{ij}^{new}=r\times (x_{ij}-x_{tj}-\gamma \times b_{ij}))+\gamma \times b_{ij}$$

#### 3.3.4. Pareto Grid Density Construction Method

In multi-objective optimization problems, individuals are judged by dominance and dense information. In this paper, a grid density construction method is used to ensure that the distribution of optimal solutions in the Pareto optimal solution set (also known as Pareto) is not too dense. The grid is a dynamic, *nGrid* bisected interval within the range of (−*inf*, +*inf*). Here, *nGrid* is a variable, which represents the number of divided grids. The value *inf* represents a number, which is far less than infinity.

The maximum and minimum values of each dimension of the median value of the nondominated solution were determined. The predefined *nGrid* was used to divide the current interval, which was divided into *nGrid* + 1. The minimum interval starts from negative infinity *inf*, and the maximum interval ends at positive infinity + *inf*, to prevent the nondominated solutions from crossing the boundary, and make the nondominated solutions fall in the grid. The formula for solving the grid index value is shown in (19). The value low*_i_* represents the minimum boundary value of the grid, and Target represents the number of objective functions. i=1,Target,Target=2,j=1,⋯,nGrid.

(19) $$grid_{ij}=\left\{\begin{array}{c} -\inf\\ low_{i}+j\times ((uper_{i}-low_{i})/nGrid)\\ \inf \end{array}\right.$$

#### 3.3.5. Pareto Optimal Solution Set Construction Method

First, constructing the optimal solution set requires a certain probability to randomly delete redundant nondominated solutions. The method to construct the deletion selection probability involves the use of the absolute value of the difference between the nondominated solution and the grid index value of the same dimension for operation. The formulas are shown in (20) and (21). The larger $poss_{i}$ the nondominated solution corresponding to Formula (20), the harder it will be to delete. The advantage of this is that the preference for a certain optimization objective brought by the nondominated solution interval is reduced, and the unified operation for all optimization objectives can be carried out fairly to obtain a relatively fair solution with the possibility of deletion.

(20) possi=∑i=1Rep∑j=1Rep∑k=1Targetgridik−gridjk

(21) $$Poss_{i}=1/(poss_{i}+1)$$

#### 3.3.6. Calculation Method of Multi-Objective Fitness

To solve the problem of the diversity of image fusion quality evaluation functions, two image fusion quality evaluation functions, cross entropy (CE) and mutual information (MI), are selected to form a multi-objective optimization problem for two objectives. The formula is shown in (22).

(22) fitness_pareto=max{CE,MI}

### 3.4. PCNN Model Structure Guided by Saliency Mechanism

**Figure 1:**
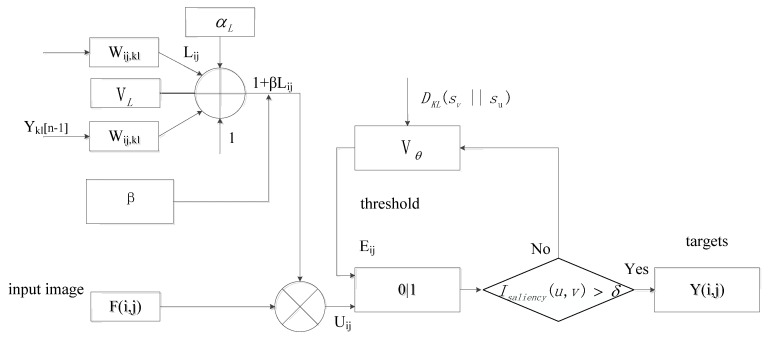
PCNN model structure guided by saliency mechanism.

The model structure is shown in Figure 1.

## 4. PCNN Transform Domain Image Fusion Method Guided by Saliency Mechanism

### 4.1. Fusion Rules

(1) Low Frequency Fusion Rules

In this paper, using the characteristics of a PCNN model’s clustering and lighting segmentation, the significance function is used as the criterion of a PCNN model’s iteration termination, and the ToF low-frequency component after an NSST decomposition is ignited and segmented. The component is recorded as $C_{ToF}^{L}$, and the low-frequency component of the color image after an NSST decomposition is recorded as $C_{RGB}^{L}$. According to the characteristics of the images collected by heterogeneous systems in the mountainous planting environment and the natural scenes of the disordered planting orchard picking operation in the Gansu Province, the low-frequency components of color images have sufficient detailed texture information, while the low-frequency components of ToF images have the characteristics of extracting targets at a certain distance and separating the background, but provide less detailed texture information. Therefore, the ToF low-frequency components and color image low-frequency components after multiple lighting segmentation using a PCNN are fused. The fusion rule uses weighted average, which is expressed as Formula (23), to highlight more foreground information belonging to the highlighted part of the ToF image.

(23) $$C_{fuse}^{L}(m,n)=0.5*C_{ToF}^{L}(m,n)+0.5*C_{RGB}^{L}(m,n)$$

(2) High frequency fusion rules

Bilateral filtering is a local, nonlinear, and noniterative technology. High-frequency fusion rules are introduced to measure the similarity between the ToF image and color image at the corresponding position of the decomposed high-frequency component, as shown in Formula (24). Let the high-frequency component of the ToF image decomposed by an NSST be $C_{ToF}^{H}$, and the high-frequency component of the color image decomposed by an NSST be $C_{RGB}^{H}$. The spatial neighborhood Gaussian function $w_{\mathrm{Neighborhood}}$ is shown in Equation (25), and the high-frequency component gray value similarity Gaussian function $w_{\mathrm{Similarity}}$ is shown in Equation (26).

(24) $$C_{fuse}^{H}(m,n)=\frac{\sum_{\left(t,s)\in Area(i,j\right)}C_{RGB}^{H}(t,s)w_{\mathrm{Neighborhood}}w_{\mathrm{Similarity}}}{\sum_{\left(t,s)\in Area(i,j\right)}w_{\mathrm{Neighborhood}}w_{\mathrm{Similarity}}}$$

(25) $$w_{\mathrm{Neighborhood}}=e^{\left(-\frac{{\left(m-t\right)}^{2}+{\left(n-s\right)}^{2}}{2\sigma^{2}}\right)}$$

(26) $$w_{\mathrm{Similarity}}=e^{\left(-\frac{{\left\|C_{ToF}^{H}(m,n)-C_{RGB}^{H}(t,s)\right\|}^{2}}{2\sigma^{2}}\right)}$$

### 4.2. Heterogeneous Image Fusion Process

**Figure 2:**
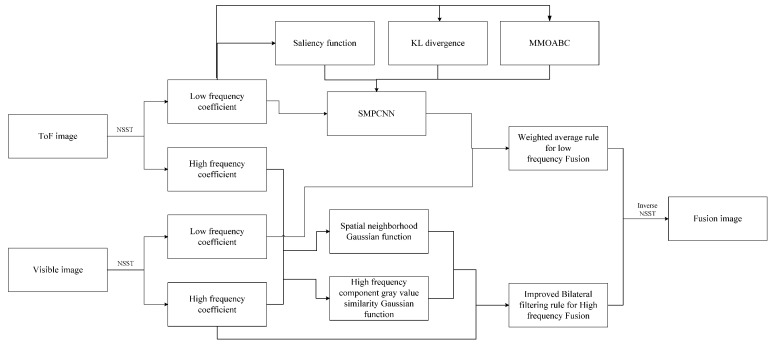
Fusion Process.

The fusion process is shown in Figure 2.

### 4.3. NSST-SMPCNN Method Multi-Source Image Fusion Steps

NSST-SMPCNN algorithm is proposed, named as Algorithm 1. The fusion steps of NSST-SMPCNN algorithm for multi-source image are as follows.
**Algorithm 1:** NSST-SMPCNN.**Input:** ToF confidence image and visible light image after registration.*M*, *N*, *u*, *v*, *n*. *limit*, *maxCycle*, *NP*, *d*, γ, *Rep*, *nGrid*, and *Target*.**Output:** Fusion image.Step 1: NSST is performed, generate u low-frequency sub-band images and 2*^v^* high-frequency sub-band images.Step 2:*n* = 1while (Equation (11))BeginCalculate the dynamic threshold amplification coefficient using Equation (12).Use Equations (13)–(22) to construct MMOABC algorithm, optimize three parameters including *α_L_*, *β* and *α_θ_*.The SMPCNN model is constructed using Equations (11)–(22).Output ignition diagram.*n ++*EndStep 3: Use Formula (23) to fuse low-frequency components with the weighted average rule; The high-frequency components are fused using the improved bilateral filter of Equations (24)–(26).Step 4: Perform NSST inverse transform.Step 5: Stop running and output the fused image.

Note: *M* and *N* represent image size, *u* represents NSST decomposition level, *v* represents NSST decomposition direction number, and *n* represents current ignition number. The maximum number of food source stagnation is *limit*, the maximum number of iterations of algorithm evolution is *maxCycle*, the number of food sources is *NP*, and the dimension of bee individual component is *d*. *γ* represents momentum, *Rep* represents the number of nondominated solutions, *nGrid* represents the number of divided grids, and *Target* represents the number of objective functions. Where, *d* = 3, *Target* = 2, *γ* = 0.9. *α_L_* represents the link channel feedback term, *β* represents link strength, and *α_θ_* represents dynamic threshold attenuation factor.

## 5. Experiment

### 5.1. Image Fusion Evaluation Index

Six models were selected for testing to evaluate the image fusion performance of the heterogeneous vision system, including a non-subsampled contourlet transform (NSCT) model [27], a fusion method for infrared and visible light images based on an NSCT (ImNSCT) [28], a DWT model [29], a simplified pulse coupled neural network (SPCNN) model [30], a single target SPCNN fusion model (ST-SPCNN) [31] and the NSST-SMPCNN model described in this paper. Nine objective image evaluation indicators [32] were selected to objectively evaluate image quality, including average gradient (AG), edge strength (ES), information entropy (IE), standard deviation (SD), peak signal to noise ratio (PSNR), spatial frequency (SF), image clarity (IC), mutual information (MI), and structural similarity (SSI). The higher the values of these nine indicators, the better the fusion image quality.

### 5.2. Public Dataset Image Fusion Experiment

In this paper, three public datasets are used for the experimental testing of heterogeneous image fusion, namely, infrared and color vineyard heterogeneous public datasets taken in natural scenes [33] and apple RGB-D image datasets published by Universitat de Lleida in Spain named fuji_apple [34,35] and PApple_RGB-D-Size [36]. The above three datasets were recorded as dataset I, dataset II and dataset III, respectively, and four groups of data in each of the three datasets were selected for testing. The results are shown in Table 1, Table 2 and Table 3, respectively. The fusion effect is shown in Table 4. The data results show that the objective evaluation indexes of the NSST-SMPCNN method described in this paper, such as AG, ES, SF, IC, and MI are the best in dataset I. For dataset II and dataset III, AG, ES, IE, SF, IC, MI, and other objective evaluation indexes of the first and fourth groups of test data are the best. The values of SD and PSNR of the five other algorithms are better than those of the algorithm in this paper. The SSI value of the DWT algorithm is the best.

### 5.3. Heterogeneous Image Fusion Experiment of Natural Orchard

In this paper, a heterogeneous vision system is established using a ToF industrial depth camera (Basler AG, Ahrensburg, Germany) and a color camera (Canon Inc., Tokyo, Japan). The ToF camera can output four types of images, including a ToF intensity image, ToF range data, ToF confidence map, and ToF point cloud image [37]. The data collection site in the natural environment is located in the experimental base of the Fruit Research Institute, Qinzhou District, Tianshui City, Gansu Province, China. More than 1000 ToF intensity images, depth images, confidence images, and color images under different lighting conditions between 10:00 and 19:00 were collected using a heterogeneous vision system. The heterogeneous images collected from the natural scene of the orchard were recorded as dataset IV, and four groups of data were selected as samples, including ToF confidence images and corresponding visible light images for testing. The results are shown in Table 5, and the fusion effect is shown in Table 4. The data results show that the NSST-SMPCNN algorithm described in this paper has the best fusion effect on the ToF confidence image and the corresponding visible light image collected in the natural scene. The values of nine indicators, including AG, ES, IE, SD, PSNR, SF, IC, MI, and SSI indicated excellent performance.

In conclusion, the experimental results show that the NSST-SMPCNN algorithm presented in this paper performs well in a test using three common datasets, as indicated by AG, ES, SF, IC, MI, and other objective evaluation indicators. This is because the significance function is used as the iteration termination condition of the PCNN model described in this paper to realize adaptive ignition termination. A new momentum-driven multi-objective artificial bee colony algorithm is used to optimize the PCNN parameters, which enhances the mechanism of the PCNN model’s gray aggregation lighting and same gray attribute priority lighting. For the dataset IV established in this paper, the NSST-SMPCNN algorithm proposed in this paper performs well in nine indicators. This shows that the weighted average rule is used to fuse the low-frequency components, which can highlight more foreground information belonging to the highlighted part in the ToF image. The high-frequency components are fused by the improved bilateral filter, which strengthens the similarity between the ToF image and the color image. The proposed NSST-SMPCNN method is suitable for heterogeneous image fusion in complex orchard environments in Gansu.

## 6. Conclusions

The traditional method of spatial domain fusion is to create a fusion model in the image gray space, which has the disadvantage of not finding the texture and boundary characteristics of the source image easily. A PCNN model has the defects of parameter experience setting, nonadaptive termination, and easy over-segmentation. This paper proposes a PCNN model guided by the saliency mechanism and applies it to the fusion of ToF and visible light heterogeneous images collected by a binocular acquisition system in an orchard environment. The iteration termination conditions and dynamic threshold amplification coefficients *V_θ_*, the feedback items of the link channel *α_L_*, link strength *β*, and dynamic threshold attenuation factor *α_θ_* are improved adaptively. A new momentum-driven multi-objective artificial bee colony algorithm (MMOABC) is used for parameter optimization. The proposed NSST-SMPCNN method combines the saliency mechanism, saliency function and PCNN clustering segmentation mechanism, and has the advantages of a grayscale clustering lighting mechanism and the same grayscale attribute first lighting, which is suitable for heterogeneous image fusion in complex orchard environments in Gansu. The data results show that the NSST-SMPCNN algorithm described in this paper has the best fusion effect on the ToF confidence image and the corresponding visible light image collected in the natural environment. The values of nine indicators, including AG, ES, IE, SD, PSNR, SF, IC, MI, and SSI, indicated excellent performance.

However, some data test results in the public dataset still have the disadvantage of a poor fusion effect, which needs further improvement. In future work, it is necessary to introduce a deep learning convolutional neural network to further explore the algorithm structure to capture better image features and improve the fusion effect.

## Figures and Tables

**Table 1:** Objective evaluation index results of dataset I.

Test Grouping	Algorithm	AG	ES	IE	SD	PSNR	SF	IC	MI	SSI	Runtime/s
First group	NSCT	8.04	78.80	7.11	108.38	14.74	19.31	10.25	1.17	0.77	42.94
	ImNSCT	7.41	71.36	7.11	110.70	14.01	17.95	9.76	1.05	0.74	53.29
	DWT	6.57	64.48	7.11	146.05	13.63	15.97	8.43	1.25	***0.79***	1.10
	SPCNN	7.41	75.69	7.15	162.28	8.19	16.34	8.75	0.02	0.32	112.76
	ST-SPCNN	8.23	80.76	7.08	***170.91***	***15.41***	19.80	10.61	0.73	0.72	91.59
	NSST-SMPCNN	***9.98***	***96.83***	***7.19***	161.13	14.17	***24.38***	***13.07***	***2.53***	0.68	60.10
Second group	NSCT	5.56	54.65	6.58	160.88	15.06	16.40	7.09	1.16	0.85	43.11
	ImNSCT	5.28	51.56	6.94	159.34	14.66	16.87	6.85	1.23	0.84	53.33
	DWT	4.18	40.99	5.71	***199.15***	15.01	13.25	5.37	1.49	***0.87***	0.87
	SPCNN	4.71	48.28	6.65	187.57	10.89	12.58	5.52	0.08	0.58	114.17
	ST-SPCNN	5.46	54.04	***7.01***	196.37	***15.79***	15.98	6.96	0.76	0.82	92.43
	NSST-SMPCNN	***6.31***	***62.02***	6.78	189.46	15.52	***19.30***	***8.07***	***2.38***	0.79	59.37
Third group	NSCT	8.49	82.45	7.10	98.47	***15.64***	20.56	10.94	1.31	0.80	43.16
	ImNSCT	7.16	68.68	6.99	91.22	14.50	18.40	9.54	1.17	0.77	53.28
	DWT	7.34	71.07	7.16	137.40	14.68	18.25	9.61	1.40	***0.82***	0.86
	SPCNN	7.64	77.76	***7.45***	151.71	7.12	16.95	9.05	0.10	0.28	114.47
	ST-SPCNN	8.08	78.10	7.25	***158.35***	15.58	20.33	10.62	0.80	0.73	90.58
	NSST-SMPCNN	***9.41***	***91.46***	7.42	149.49	14.52	***23.43***	***12.31***	***2.72***	0.74	59.08
Fourth group	NSCT	6.35	62.86	6.69	127.60	14.26	17.19	8.00	1.22	***0.78***	43.20
	ImNSCT	6.31	62.48	6.67	105.31	12.28	18.40	8.04	1.03	0.71	53.22
	DWT	5.08	50.87	6.68	156.04	13.71	13.73	6.35	1.14	0.78	0.87
	SPCNN	6.17	64.08	***7.21***	146.58	6.82	14.95	7.11	0.11	0.38	113.38
	ST-SPCNN	6.64	66.04	7.15	153.50	***15.23***	17.87	8.40	1.03	0.72	94.33
	NSST-SMPCNN	***7.01***	***69.04***	6.90	***176.65***	12.30	***20.05***	***8.94***	***2.60***	0.70	61.12

**Table 2:** Objective evaluation index results of dataset II.

Test Grouping	Algorithm	AG	ES	IE	SD	PSNR	SF	IC	MI	SSI	Runtime/s
First group	NSCT	8.80	94.41	7.31	96.34	13.59	19.61	9.53	1.15	0.55	51.96
	ImNSCT	10.00	107.21	7.48	***125.04***	12.21	22.56	10.86	1.00	0.49	50.65
	DWT	8.11	88.06	7.54	90.38	14.12	17.29	8.58	1.34	***0.63***	0.94
	SPCNN	9.14	99.34	6.43	54.12	2.45	20.98	9.68	0.08	0.11	106.01
	ST-SPCNN	9.52	102.86	6.36	53.90	12.39	22.74	10.22	0.62	0.47	85.08
	NSST-SMPCNN	***11.38***	***122.06***	***7.80***	114.76	***14.27***	***26.18***	***12.42***	***2.48***	0.57	68.69
Second group	NSCT	***7.81***	***83.89***	7.34	***90.94***	13.98	16.27	***8.43***	0.98	0.52	51.01
	ImNSCT	7.14	76.68	7.30	89.15	14.21	15.54	7.76	0.90	0.49	50.14
	DWT	5.45	59.25	7.15	61.89	***18.76***	10.97	5.75	1.14	***0.66***	0.9
	SPCNN	7.05	76.64	6.81	52.55	2.52	14.69	7.43	0.13	0.13	104.93
	ST-SPCNN	7.23	78.30	6.86	54.76	17.55	15.18	7.69	0.79	0.61	84.15
	NSST-SMPCNN	7.41	78.84	***7.35***	84.95	13.51	***16.56***	8.18	***2.05***	0.55	64.48
Third group	NSCT	***8.54***	***91.39***	7.55	116.39	11.89	***19.58***	***9.28***	1.04	0.56	52.29
	ImNSCT	7.59	81.14	7.36	119.16	10.53	18.02	8.29	0.88	0.52	49.77
	DWT	6.07	65.73	7.46	87.39	***12.55***	13.40	6.45	1.17	***0.64***	0.93
	SPCNN	7.78	84.38	6.99	58.63	2.29	17.71	8.22	0.04	0.14	105.81
	ST-SPCNN	8.19	88.54	7.27	71.33	11.07	18.59	8.72	0.61	0.55	85.24
	NSST-SMPCNN	8.29	88.02	***7.59***	***126.49***	8.99	19.48	9.17	***2.10***	0.50	64.03
Fourth group	NSCT	11.93	127.80	7.65	130.21	***11.27***	25.14	12.95	1.13	0.53	51.34
	ImNSCT	11.17	119.45	7.49	***132.18***	9.60	23.56	12.15	0.94	0.50	49.97
	DWT	8.41	91.16	7.51	91.92	10.02	17.15	8.95	1.35	***0.56***	0.91
	SPCNN	9.35	101.56	6.50	52.41	2.50	20.53	9.90	0.08	0.11	103.57
	ST-SPCNN	9.52	103.07	6.47	53.10	9.30	21.11	10.14	0.61	0.44	83.84
	NSST-SMPCNN	***12.06***	***128.26***	***7.83***	127.82	10.21	***26.26***	***13.34***	***2.25***	0.50	62.62

**Table 3:** Objective evaluation index results of dataset III.

Test Grouping	Algorithm	AG	ES	IE	SD	PSNR	SF	IC	MI	SSI	Runtime/s
First group	NSCT	7.36	71.32	7.04	112.62	15.49	22.77	9.46	1.02	0.72	43.89
	ImNSCT	6.34	56.88	6.71	119.29	15.05	23.82	9.23	0.78	0.70	55.02
	DWT	4.79	46.61	6.80	122.30	***15.60***	16.55	6.30	1.77	***0.73***	0.96
	SPCNN	5.41	55.79	5.97	***127.07***	15.20	21.20	6.48	***1.84***	0.69	117.29
	ST-SPCNN	7.66	73.53	6.68	89.06	14.73	***27.12***	***10.40***	0.42	0.67	93.79
	NSST-SMPCNN	***7.90***	***81.41***	***7.16***	116.07	4.99	19.36	9.20	0.00	0.26	62.61
Second group	NSCT	***9.06***	***88.94***	***7.16***	143.25	10.00	25.23	***11.45***	0.80	0.63	43.66
	ImNSCT	7.63	69.52	6.83	136.04	9.39	25.90	10.83	0.57	0.63	55.61
	DWT	5.25	51.77	6.70	158.34	9.99	16.45	6.76	***1.69***	***0.67***	0.94
	SPCNN	7.05	76.64	6.81	52.55	2.52	14.69	7.43	0.13	0.13	117.76
	ST-SPCNN	8.55	82.82	6.92	98.36	***10.40***	27.76	11.41	0.34	0.59	94.81
	NSST-SMPCNN	7.14	67.16	6.02	***211.49***	6.39	***38.28***	9.94	1.58	0.47	63.42
Third group	NSCT	***8.00***	***81.02***	***7.08***	106.87	***13.60***	20.54	***9.60***	0.86	0.67	46.2
	ImNSCT	6.57	64.20	6.69	108.21	12.48	18.48	8.52	0.62	0.66	55.79
	DWT	4.51	45.92	6.65	100.75	12.83	12.18	5.47	1.60	***0.71***	0.96
	SPCNN	6.21	64.69	6.55	66.75	2.55	15.22	7.06	0.00	0.22	116.82
	ST-SPCNN	6.87	68.94	6.82	80.91	11.39	19.64	8.59	0.43	0.66	93.99
	NSST-SMPCNN	6.82	67.30	5.82	***133.85***	9.39	***29.88***	8.86	***1.73***	0.53	62.89
Fourth group	NSCT	7.64	78.40	***7.34***	128.74	***10.68***	19.09	8.95	1.00	0.65	44.07
	ImNSCT	6.34	62.94	7.02	118.23	8.58	17.25	8.04	0.78	0.65	55.75
	DWT	4.45	46.05	7.00	110.58	8.75	11.57	5.23	***1.91***	***0.70***	0.97
	SPCNN	5.88	61.87	6.73	63.67	2.41	13.89	6.56	0.00	0.22	117.76
	ST-SPCNN	6.38	64.84	6.70	60.88	8.96	17.19	7.75	0.33	0.57	93.13
	NSST-SMPCNN	***11.54***	***107.64***	5.88	***174.90***	2.65	***63.36***	***16.44***	1.79	0.41	62.43

**Table 4:** Fusion effect of four datasets.

Dataset	Test Grouping	NSCT	ImNSCT	DWT	SPCNN	ST-SPCNN	NSST-SMPCNN
dataset I	First group	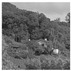	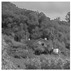	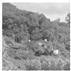	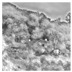	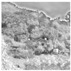	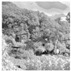
	Second group	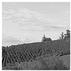	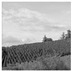	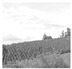	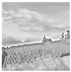	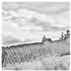	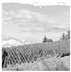
	Third group	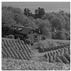	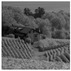	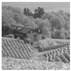	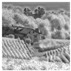	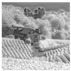	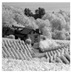
	Fourth group	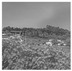	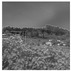	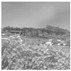	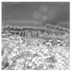	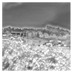	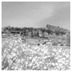
dataset II	First group	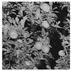	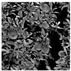	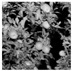	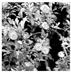	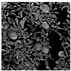	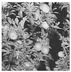
	Second group	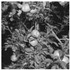	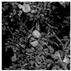	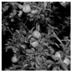	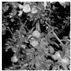	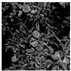	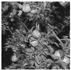
	Third group	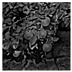	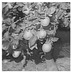	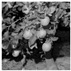	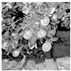	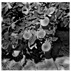	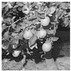
	Fourth group	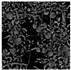	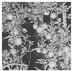	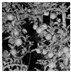	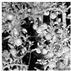	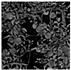	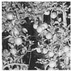
dataset III	First group	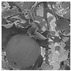	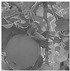	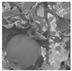	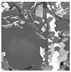	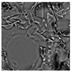	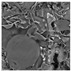
	Second group	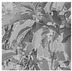	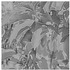	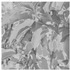	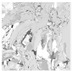	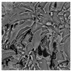	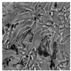
	Third group	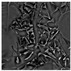	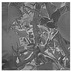	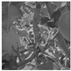	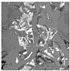	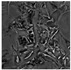	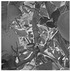
	Fourth group	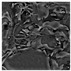	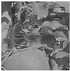	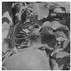	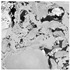	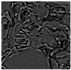	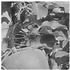
dataset IV	First group	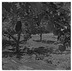	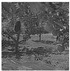	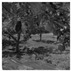	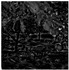	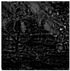	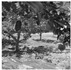
	Second group	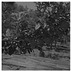	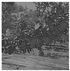	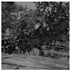	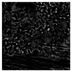	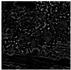	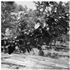
	Third group	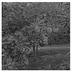	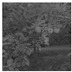	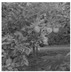	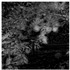	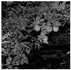	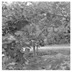
	Fourth group	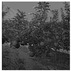	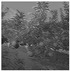	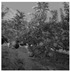	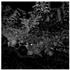	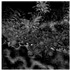	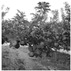

**Table 5:** Objective evaluation index results of dataset IV.

Test Grouping	Algorithm	AG	ES	IE	SD	PSNR	SF	IC	MI	SSI	Runtime/s
First group	NSCT	8.87	86.29	6.78	70.67	12.90	22.79	11.41	0.74	0.49	42.92
	ImNSCT	10.36	99.70	6.89	91.08	13.02	27.73	14.02	0.54	0.50	52.60
	DWT	7.66	74.24	6.85	75.18	13.03	21.35	10.22	1.32	0.49	1.05
	SPCNN	9.74	98.90	6.80	53.19	2.15	24.57	11.66	0.01	0.08	111.08
	ST-SPCNN	9.75	94.74	5.99	32.68	13.30	28.72	12.91	0.29	0.33	90.74
	NSST-SMPCNN	***12.05***	***116.21***	***7.57***	***111.20***	***18.94***	***32.60***	***16.17***	***2.39***	***0.54***	61.39
Second group	NSCT	9.01	88.30	6.88	70.64	11.88	23.30	11.64	0.92	0.46	42.96
	ImNSCT	10.96	105.58	7.02	94.91	12.20	29.39	14.99	0.69	0.48	53.08
	DWT	8.07	78.12	6.97	73.39	12.04	22.69	10.91	1.77	0.45	0.91
	SPCNN	8.44	86.34	5.44	24.33	1.80	23.49	10.08	0.01	0.04	115.60
	ST-SPCNN	9.74	95.15	5.58	25.72	12.31	29.59	13.01	0.23	0.26	90.66
	NSST-SMPCNN	***13.16***	***127.12***	***7.78***	***116.50***	***20.47***	***35.31***	***17.85***	***2.69***	***0.52***	62.22
Third group	NSCT	7.99	78.46	6.58	81.35	13.71	20.00	9.56	0.48	0.53	43.00
	ImNSCT	5.32	49.74	6.22	78.15	13.78	14.64	6.46	0.38	0.45	52.86
	DWT	6.63	65.81	6.76	100.27	14.13	17.08	8.09	0.69	***0.55***	0.91
	SPCNN	8.47	86.13	6.82	52.13	1.81	21.03	9.53	0.00	0.10	114.72
	ST-SPCNN	8.88	87.54	6.89	54.09	13.90	***22.10***	11.11	0.39	0.39	88.77
	NSST-SMPCNN	***8.98***	***88.85***	***7.10***	***129.25***	***17.66***	22.07	***11.46***	***2.22***	0.53	60.59
Fourth group	NSCT	7.01	68.99	6.61	73.43	11.73	18.97	8.81	0.78	0.49	42.97
	ImNSCT	8.09	78.21	6.78	93.76	11.72	22.85	10.70	0.61	0.49	55.65
	DWT	6.33	62.38	6.74	88.30	12.00	18.63	8.19	1.12	0.51	0.91
	SPCNN	8.14	83.60	6.58	41.47	1.41	21.13	9.55	0.00	0.08	113.80
	ST-SPCNN	8.58	84.80	6.35	37.43	12.83	25.50	11.06	0.37	0.38	90.20
	NSST-SMPCNN	***9.51***	***93.20***	***7.05***	***127.49***	***16.31***	***27.43***	***12.36***	***2.29***	***0.53***	59.78

## Data Availability

The datasets generated and/or analyzed during the current study are available from the corresponding author on reasonable request.
